# Alcohol-based hand sanitisers as first line of defence against SARS-CoV-2: a review of biology, chemistry and formulations

**DOI:** 10.1017/S0950268820002319

**Published:** 2020-09-29

**Authors:** D. Singh, K. Joshi, A. Samuel, J. Patra, N. Mahindroo

**Affiliations:** 1 School of Health Sciences, University of Petroleum and Energy Studies, Energy Acres, Bidholi, Via Premnagar, Dehradun 248007, Uttarakhand, India; 2 Department of Biotechnology, BJM School of Biosciences, Indian Institute of Technology Madras, Chennai, 600036, India; 3 Department of Morphology, Surgery and Experimental Medicine, Universita'Degli Studi di Ferrara, Via Savonarola, 9, 44121 Ferrara, FE, Italy

**Keywords:** Alcohol rubs, COVID-19, encapsulated viruses, ethanol, hand sanitiser, isopropanol, SARS-CoV-2

## Abstract

The pandemic due to Severe Acute Respiratory Syndrome Coronavirus 2 (SARS-CoV-2) has emerged as a serious global public health issue. Since the start of the outbreak, the importance of hand-hygiene and respiratory protection to prevent the spread of the virus has been the prime focus for infection control. Health regulatory organisations have produced guidelines for the formulation of hand sanitisers to the manufacturing industries. This review summarises the studies on alcohol-based hand sanitisers and their disinfectant activity against SARS-CoV-2 and related viruses. The literature shows that the type and concentration of alcohol, formulation and nature of product, presence of excipients, applied volume, contact time and viral contamination load are critical factors that determine the effectiveness of hand sanitisers.

## Background

The outbreak of respiratory infection with Severe Acute Respiratory Syndrome Coronavirus 2 (SARS-CoV-2) virus has emerged as a serious global public health threat [1]. It is the third time in the last two decades that an animal coronavirus has emerged to cause epidemic infection in humans. The disease was first reported in Wuhan province of China at the end of 2019 but rapidly spread to infect more than 23 million people as of 25 August 2020, and has been associated with > 800 000 deaths [2].

The World Health Organization (WHO) declared a pandemic on 11 March 2020 and the infection has spread across almost all countries and regions of the world. Most infections appear to be asymptomatic or with mild flu-like symptoms but severe and life-threatening presentations including pneumonia, fever, nausea and gastrointestinal upset have been associated with individuals with predisposing factors, particularly age, respiratory insufficiency, diabetes and obesity, among others [3]. The WHO, and national disease control agencies, have continuously emphasised the importance of hand hygiene to reduce spread of the virus. WHO guidelines recommend maintaining hand hygiene, by frequent washing using soap and water for at least 20 s especially after going to the bathroom, before eating and after coughing, sneezing or blowing one's nose. When soap and water are not available, the Food and Drug Administration (FDA) recommends sanitising of non-visibly soiled hands with an alcohol-based agent containing 80% v/v ethanol or 75% v/v isopropanol [4].

Enveloped viruses such as coronavirus and influenza A H1N1 are able to survive on inanimate surfaces for long periods [5]. It has been reported that some COVID-19 patients discharged the virus in their stool for up to 73 days after symptom onset [6], and as diarrhoea is a common symptom, faecal to oral cross-transmission is likely [7], and hence maintaining effective hand hygiene is paramount.

Alcohol-based hand sanitisation is widely considered to be effective to reduce or eliminate bacterial/viral load, but with variable compliance rates [8]. The alcohols, ethanol, isopropanol and *n-*propanol as used for disinfection are commonly applied in the form of hand rub rinses, gels and foams.

Owing to the increasing demand for hand sanitisation to control the spread of SARS-CoV-2, some manufacturers have resorted to their own formulations, which are not validated and licensed for use. To combat this, the FDA, WHO, the United States Pharmacopeia (USP) and the Central Drugs Standard Control Organization (CDSCO), India, have produced guidelines for the formulation and manufacture of such preparations [4, 9, 10]. This review assesses available information on the composition, formulation and effectiveness of alcohol-based hand disinfection products with specific reference to their activity against SARS-CoV-2.

## Structural features of SARS-CoV-2

**Fig. 1.**
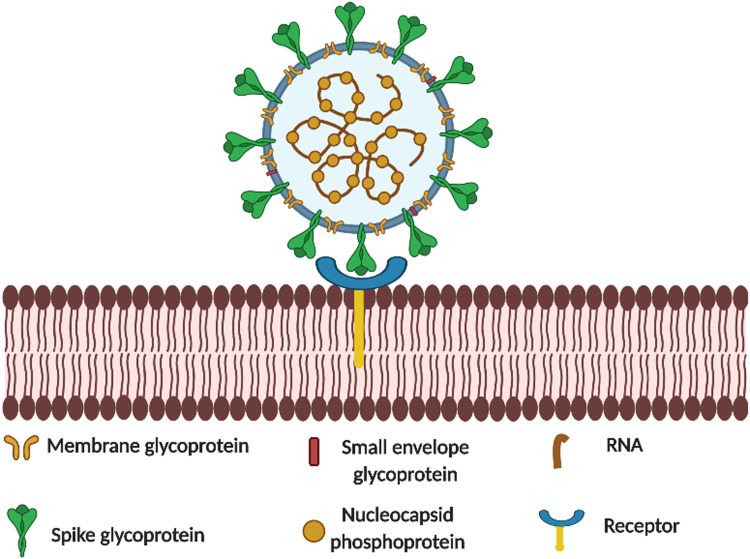
Binding of SARS-CoV-2 to ACE-2 receptor.

SARS-CoV-2 is a new member of the family *Coronaviridae*, order *Nidovirales*, and comprise of two sub-families, *Coronavirinae* and *Torovirinae* [11]; it is the seventh coronavirus known to infect humans [12]. SARS-CoV-2 is relatively large in size (0.12 μm) and characterised by the presence of highly glycosylated spikes on the protein membrane in a crown-like arrangement, hence the name, Corona (Fig. 1). It has a single-stranded positive-sense RNA genome of 29 891 nucleotides. The glycosylated spike protein binds to the host angiotensin converting enzyme-2 (ACE-2) protein which serves as a functional receptor for entry into host respiratory cells. This receptor also binds the earlier SARS-CoV but with 10–20 times less affinity than for SARS-CoV-2 spike protein [13, 14].

## Chemistry of virucidal action of hand sanitisers

**Fig. 2.**
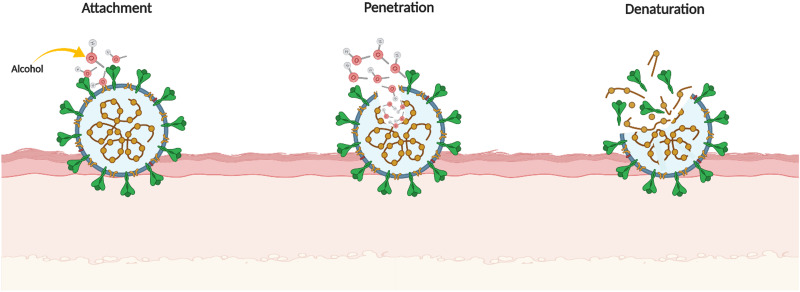
Antiviral mechanism of action of alcohol against enveloped viruses.

Several antimicrobial compounds have been utilised for hand disinfection and include, among others, alcohols, chlorhexidine, chloroxylenol, hexachlorophene, benzalkonium chloride, cetrimide, triclosan and povidone-iodine [15]. The alcohols, namely ethanol and isopropanol, are most commonly used for skin disinfection due to their broad activity against bacteria, viruses and fungi [16]; their mode of action against enveloped viruses is shown in Figure 2.

Lipid membrane dissolution and protein denaturation are key mechanisms of the antimicrobial action of ethanol, leading to the disruption of membrane and inhibition of metabolism [17, 18].

Alcohols are amphiphilic compounds, as they possess both hydrophilic and lipophilic (hydrophobic) properties that facilitate their entry through the viral envelope. The outermost membrane of SARS-CoV-2 comprises lipids bound together by an alkane chain of hydrophobic fatty acids. Contact of the virus with an alcohol leads to alteration in its membrane fluidity [19]. The presence of polar oxygen atoms weaken the lipophilic interactions between the non-polar residues, and increase the internal affinity of the membrane for water, thus destabilising and denaturing the protein structure [17]. The antimicrobial mechanism of alcohol against enveloped viruses is similar to that for bacteria as both have a lipid-rich outer membrane. Non-enveloped viruses are relatively more resistant to this mechanism due to the lack of a lipid membrane.

## Viruses similar to SARS-CoV-2

The family *Coronaviridae* is comprised of four groups (Table 1). SARS-CoV-2 is considered to be taxonomically related to group 2 coronaviruses [20, 21]. Virus and bovine viral diarrhoea virus (BVDV) are used for testing the effectiveness of chemical disinfectants and antiseptics against enveloped viruses according to DVV/Robert Koch Institute (RKI) guidelines [22]. The Modified Vaccinia Ankara (MVA) virus can also be used as a surrogate model for this purpose as it exhibits high stability against alcohol-based inactivation. The latter virus does not replicate in humans, thus eliminating the risk of disease through unintentional inoculation [23, 24]. Bovine coronavirus (BCV) has been used as a surrogate virus for SARS-CoV [25], and owing to its high (80%) relatedness to SARS-CoV-2, consequently may have potential value as a surrogate test agent for the latter.

**Table 1.** Classification of Coronaviruses

Groups	Species
Group 1 *α*-CoVs	Transmissible gastroenteritis coronavirus (TGEV)
	Canine coronavirus (CCoV)
	Porcine respiratory coronavirus (PRCoV)
	Feline coronavirus (FeCoV)
	Porcine epidemic diarrhoeal coronavirus (PEDV)
	Human coronavirus 229E (HCoV-229E)
	Human coronavirus NL63 (HCoV-NL63)
Group 2 *β*-CoVs	Bat coronavirus (BCoV)
	Porcine haemagglutinating encephalomyelitis virus (HEV)
	Murine hepatitis virus (MHV)
	Human coronavirus 4408 (HCoV-4408)
	Human coronavirus OC43 (HCoV-OC43)
	Human coronavirus HKU1 (HCoV-HKU1)
	Severe acute respiratory syndrome coronavirus (SARS-CoV)
	Middle Eastern respiratory syndrome coronavirus (MERS-CoV)
Group 3 *γ*-CoVs	Avian infectious bronchitis virus (IBV)
	Turkey coronavirus (TCoV)
Group 4 *δ*-CoVs	Bulbul coronavirus HKU11
	Thrush coronavirus HKU12
	Munia coronavirus HKU13

## Guidelines for testing of hand-disinfecting agents

The two most widely used guidelines for testing and regulation of hand disinfectants are the European Committee for Standardization (CEN) and the Food and Drug Administration (FDA), according to standards set by the American Society for Testing and Materials (ASTM).

### CEN standards

EN 1499 and EN 1500 are the standard methods related to hygienic hand wash and hygienic hand disinfection respectively [26, 27]. In EN 1499, agents are tested against a reference non-medicated soap and in EN 1500 against 60% v/v isopropanol, both applied for 1 min. In the latter standard, the test hand rub formulation should not be significantly inferior, in terms of log reduction of the challenge microbe, compared with the reference alcohol-based product.

EN 14476 is the standard method for evaluating the virucidal activity of disinfectants [28] and is based on an *in*-*vitro* quantitative suspension test in which agents should exhibit a minimum of 4-log reduction in viability of the microbe. Poliovirus, adenovirus and murine norovirus serve as the basis for efficacy evaluation of surface disinfectants.

prEN 16777 is also a quantitative virucidal test method and is recommended for nonporous surfaces (*in*-*vivo* carrier test); a 4-log reduction is specified and ready-to-use surface disinfectants should be tested undiluted using adenovirus and murine norovirus as test pathogens. This test method simulates practical conditions and together with EN 14476 forms the basis for biocidal product registration in Europe [29].

### ASTM standards

#### ASTM E-1838

A finger pad test method designed to compare the virus-eliminating effectiveness of hand washing and hand rubbing sanitisers using at least three healthy participants. Exposure time should be 10–20 s for hand washing and 20–30 s for a hand sanitation. The recommended test viruses include adenovirus 5, feline calicivirus, rotavirus, rhinovirus and murine norovirus at a minimum of 10^4^ infectious units with or without a soil load. A 4-log reduction in virus load must be demonstrated by the test product in the presence and absence of 5% foetal bovine serum [30].

#### ASTM E-2011

This method evaluates the virucidal activity of hand wash and hand rub agents against viruses and is claimed to better reflect actual working conditions as it incorporates mechanical friction during whole-hand decontamination. At least three healthy participants are required and following application of virus suspension, the specified product exposure times are 10–20 s for hand washing and 20–30 s for a sanitiser. Test viruses include adenovirus type 2 or 5, feline calicivirus, rotavirus, rhinovirus and murine norovirus in the presence and absence of 5% foetal bovine serum as an interfering substance to simulate dirty conditions [31, 32].

#### ASTM E-2197

This method determines the efficacy of test disinfectants to inactivate viruses on disk carriers of brushed stainless steel, which act as a surrogate material for hard, non-porous environmental surfaces and medical devices [33, 34]

### German standards

The German Society for Control of Viral Diseases (DVV) and Robert Koch-Institute (RKI) [35] guidelines for quantitative virucidal tests on non-porous surfaces. Recommended test agents include the elstree vaccinia strain, poliovirus vaccination strain type I, LSc-2ab strain, adenovirus type 5 and polyomavirus (formerly, papovavirus) SV 40 strain 777.

#### DVV/RKI suspension test

This test is designed to determine activity against enveloped viruses, namely bovine diarrhoeal, and vaccinia viruses. The minimum test range for activity against all viruses is murine norovirus, adenovirus, poliovirus, polyomavirus and SV40 with a 4-log reduction in the presence and absence of 10% foetal calf serum [36].

#### DVV carrier test

This test is required to verify activity against vaccinia virus. The minimum test spectrum for all viruses is classified at two levels: (a) low level – vaccinia virus, murine norovirus and adenovirus and (b) high level – adenovirus, murine norovirus and murine parvovirus, with a minimum 4-log reduction in the presence and absence of 10% foetal calf serum [36].

## Alcohol type and concentration

Most alcohols exhibit a broad spectrum of germicidal activity against vegetative bacteria, viruses and fungi. In general, isopropanol is considered to have better activity against bacteria, while ethanol is more potent against viruses. However, the degree of effect depends on the percentage concentrations of the alcohol and the physical properties of the target microorganism. Isopropanol is more lipophilic than ethanol and is consequently less active against hydrophilic viruses such as polioviruses. Being a lipophilic enveloped virus, SARS-CoV-2 exhibits greater susceptibility to isopropanol than ethanol [20, 37, 38].

The optimum bactericidal concentrations of alcohols range from 60% to 90% v/v solutions in water but are generally ineffective against most microorganisms below 50% v/v [39]. The effect of different concentrations of alcohol against enveloped viruses is shown in Table 2 [25, 37, 40–46]. A recent study has shown that >30% concentrations of ethanol or isopropanol were effective in inactivating SARS-CoV-2 within 30 s [47]. Propanol has a marginally higher boiling point than ethanol, hence, the drying time of isopropanol is slightly longer compared to ethanol [48].

**Table 2.** Effect of alcohol type and concentration (% v/v)

Alcohol type and concentration	Test virus	Log_10_ reduction factor	Reference
Ethanol (62%) foam	H1N1 virus	⩾3.2	40
Ethanol (62%) gel	H1N1 virus	⩾3.2	40
Ethanol (65.9%) containing wipe	H1N1 virus	⩾3.2	40
Ethanol (62%) gel	Transmissible gastroenteritis virus	4	41
Ethanol (70%)	Mouse hepatitis virusTransmissible gastroenteritis virus	3.9 3.2	41
Ethanol (70%)	Mouse hepatitis virus Canine coronavirus	4 3.2	42
Ethanol (70%)	Human coronavirus 229E	⩾3	42
Ethanol (70%)	Respiratory syncytial virus	⩾5	43
Ethanol (70%)	SARS-CoV	Reduction under detection limits	44
Ethanol (71%)	Transmissible gastroenteritis virus	3.5	42
Ethanol (78%)	SARS-CoV	⩾5.01	45
Ethanol (80%)	Bovine viral diarrhoea virus Vaccinia virus	4.6 4.9	37
Ethanol (80%)	SARS-CoV	⩾4.2	46
Ethanol (85%) gel	SARS-CoV	⩾5.5	46
Ethanol (95%)	SARS-CoV	⩾5.5	46
Isopropanol (50%)	Mouse hepatitis virus Canine coronavirus	3.7 3.7	42
Isopropanol (70%)	SARS-CoV	⩾3.31	45
Isopropanol (45%) + *n-*propanol (30%)	SARS-CoV	⩾5.01	45
Isopropanol (45%) + *n-*propanol (30%)	SARS-CoV	⩾4.2	46
Ethanol (55%) + *n-*propanol (10%)	Bovine coronavirus	⩾4	25

## WHO formulations for hand disinfection

The WHO has recommended two alcohol-based hand sanitiser formulations which differ only in their alcohol constituent, and is widely followed throughout the world.

*Formulation 1*: Ethanol 80% v/v, glycerol 1.45% v/v, hydrogen peroxide (H_2_O_2_) 0.125% v/v.

*Formulation 2*: Isopropyl alcohol 75% v/v, glycerol 1.45% v/v, hydrogen peroxide 0.125% v/v [49].

Due to the inherent variability of raw materials and the volatility of alcohol, and in response to the COVID-19 pandemic, the United States Pharmacopeia has issued a revision of WHO formulation 2 by increasing the concentration of isopropanol to 91% v/v [10]. An *n-*propanol-based formulation has not been proposed owing to the lack of safety data on human use [49]. In March 2020, the FDA recommended the industry to use either of the two WHO formulas but emphasised that ethanol should not be used at a concentration of <94.9% by volume. In a separate FDA guideline addressing the preparation and distribution of alcohol for incorporation in hand disinfectants, mention was made of the search for other active constituents including the use of denaturants such as acetone [50]. There was also comment that the recommended amount of glycerol in the WHO formulation might negatively impact the effectiveness of isopropanol [50]. Nevertheless, both WHO formulations have been shown to be effective against SARS-CoV-2 [47]. Indeed, with regards to the latter, CDC recommends the use of alcohol-based sanitisers containing >60% ethanol or 70% isopropanol for personnel working in healthcare settings [51]. This is supported by the finding that the WHO formulation containing isopropanol had higher activity against enveloped viruses [52].

## Factors influencing the effectiveness of sanitisers against SARS-CoV-2

**Fig. 3.**
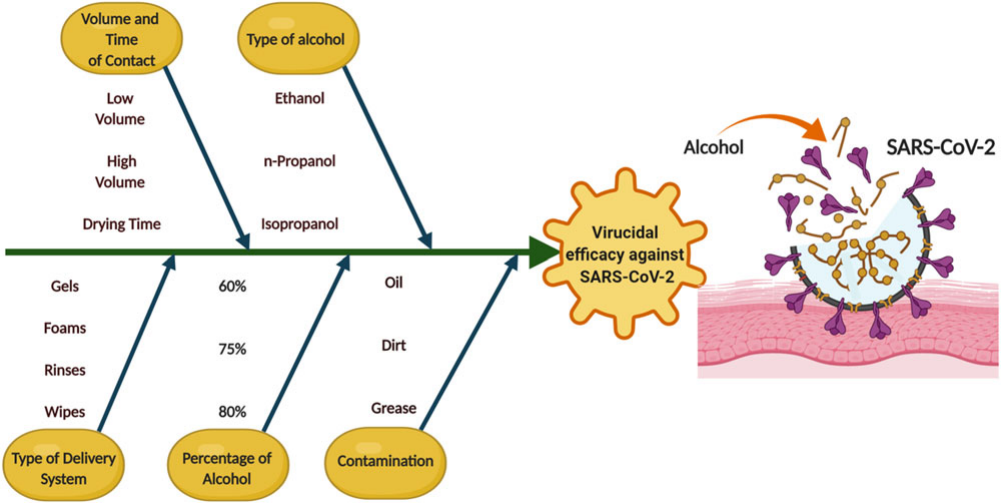
Factors affecting the efficacy of alcohol-based hand sanitisers against SARS-CoV-2.

The virucidal efficacy of hand sanitisers depends on several factors. As illustrated by the Ishikawa diagram (Fig. 3) showing the key factors which determine the efficacy of alcohol against SARS-CoV-2.

### Formulation

The most commonly used formulations for hand sanitisers are rinse, foam, gel, wipes and spray. The 70% ethanol-based liquid products have proved highly effective against the non-enveloped viruses, poliovirus and adenovirus following exposure for 30 s [53]. Alcohol-based hand rubs in the form of foam, rinse and gel did not differ significantly in trials of antimicrobial activity but the application volume and drying time had a profound effect on their efficacy [54]. Another study, however, found that alcohol-based hand wipes were comparable in activity to foam and gel products against enveloped influenza (H1N1) virus. This was ascribed to better mechanical friction achieved with wipes, resulting in additional physical removal of virus that might survive the antimicrobial treatment [40]. Indeed, another comparative study concluded that hand gels are less effective for hand hygiene because of a shorter application time (<30 s) and therefore should not replace alcohol-based liquid hand disinfectants, or used as first choice agents [55] despite the benefit of reducing skin irritation and dryness associated with liquid alcohol agents preparations. However, gel preparations containing 62% ethanol have been reported to be superior to 70% ethanol for the inactivation of surrogate coronaviruses MHV and TGEV on hard surfaces [41].

Foams have an advantage of better compliance by users due to ease of handling, non-spilling and non-stickiness. Bis-PEG12-dimethicone is commonly used as the foaming agent. It is recommended that an amount equivalent in size to a golf ball should be applied to hands [56]; they also have the added benefit of the shortest drying times compared with rinses and gels [57]. The approximate drying times of different alcohol-based formulations are given in Table 3.

**Table 3.** Effect of volume, type of alcohol-based formulation and drying time for hand disinfection

Alcohol	Drying time (in s)		
Volume and type of alcohol	Gel	Foam	Rinse
1.5 ml of 80% ethanol	27	19	24
3 ml of 80% ethanol	44	35	35
1.5 ml of 60% isopropanol	31	26	27
3 ml of 60% isopropanol	63	46	46

### Volume and contact time

An increase in the volume of alcohol and contact time results in increased efficacy of alcohol-based hand sanitisers. One pump dispenser push releases approximately 1.5 ml of gel containing 70% alcohol has been found to be insufficient for complete coverage of both hands and hence, do not comply with ASTM efficacy standards [57]. The use of 3 ml volume for foam, rinse and gel sanitisers containing 70%, 80% and 90% alcohol, respectively, is necessary to meet EN 1500 efficacy requirements, but the drying times of all preparations exceeded 30 s [54]. The amount of sanitiser used also depends on the size of the subject's hands; females are relatively smaller (mean of eight volunteers 148.39 cm^2^, RSD = 5.17), and a lower volume of the agent could be sufficient when compared with men's hands (mean of eight volunteers 183.63 cm^2^, RSD = 7.5) [58]. It is generally acknowledged that the ideal application volume is unknown, but US national guidelines suggest that a drying time of <15 s is insufficient [59], while the WHO recommends use of a ‘palmful’ of product and that the hand-hygiene process should take at least 20 s [60]. Rotter *et al*. found that 3 ml of the EN 1500 reference product (isopropanol) takes more than 49 s to dry, despite a specified rub-time of 30 s [61]. Similarly, a trial on disinfection of volunteer hands artificially contaminated with *Escherichia coli* K12 showed that WHO formulations containing either ethanol or isopropanol did not comply with the EN 1500 requirement as 60 s were taken to achieve the required log reduction. This led to the proposal that the ethanol concentration should be changed from 80% v/v to 80% w/w (equivalent to 85% v/v), and for isopropanol from 75% v/v to 75% w/w (equivalent to 80% v/v) [62]. The contact time of the agent is also relevant as a survey showed that the majority of nursing staff took only 6–24 s for hand cleansing [63]. It has also been suggested that better compliance might be achievable in the hospital setting through listening to background music during the process [64].

### Excipients

Glycerin is added in hand sanitisers as a humectant to reduce loss of skin moisture. WHO-recommended formulations contain glycerin but other nontoxic or allergenic emollients miscible in water and alcohol are not permitted for skin care [49].

Studies have shown that glycerol can reduce the efficacy of isopropanol-based sanitiser through agglomerates of flaking skin cells forming in the sticky glycerol [65]. A mixture of ethylhexylglycerin, dexpanthenol and a fatty alcohol serves as a suitable alternative with no effect on hand rub efficacy [66]. Indeed, the removal of glycerol from a formulation markedly increased the bactericidal activity of an isopropanol-based sanitiser [67]. This negative impact of glycerol has been noted in FDA guidelines regarding temporary compounding of alcohol-based hand sanitisers by industry during the COVID-19 pandemic [4]. Similarly, reducing the glycerol content from 1.45%, as per the WHO formulation, to 0.5% provided a better balance between antimicrobial efficacy and skin tolerance [68]. An extract of the Aloe vera plant has also been used as an emollient [69].

### pH

Human and canine corona viruses are reported to be more stable at a slightly acidic than alkaline pH [70, 71] but mild alkaline (pH 8) conditions are sufficient to induce conformational changes in the spike protein of coronavirus mouse hepatitis virus [72]. Both high and low pH cause inactivation of SARS-CoV [73]. The virucidal activity of ethanol against poliovirus and MS2 phage is significantly increased on the addition of sodium hydroxide [74] due to protein denaturation [75]. Sodium hydroxide has also been shown to have cidal activity against surface dried lipid enveloped human immunodeficiency virus (HIV), bovine diarrhoeal virus and pseudorabies virus [76]. Other anti-viral agents include acetic acid and calcium hydroxide against influenza virus on hard and non-porous surfaces [75]. Moreover, citric acid and urea (2%) have been reported to increase the effectiveness of alcohol-based sanitisers [37]; citric and malic acid, in combination with 70% alcohol have also been suggested to enhance killing of rhinovirus on hands [77].

### Dirt and soil contamination

It is quite likely that the effect of hand sanitisers is reduced in the presence of dirt or soil on hands. A number of interfering substances have been used to simulate dirty conditions including foetal calf serum, bovine serum albumin and sheep erythrocytes according to DVV, RKI, ASTM and CEN standard guidelines [37, 78]. Soap hand wash coupled with an alcohol gel sanitiser was shown to be more effective than either agent used alone, and activity persisted for longer [79]. These findings are corroborated by other studies showing increased reduction of murine norovirus with a wash-sanitiser regimen compared to washing with 70% ethanol alone in the presence of a high level of organic loads [80]. However, it is worth noting that hand washing with soap and water alone was found to be more effective than alcohol-based rubs for hands soiled with meat [81].

## Conclusion

Hand hygiene by washing hands with soap and water or with alcohol-based hand sanitisers are primary preventive measures against the spread of SARS-CoV-2. This review of the literature shows that several factors are pertinent to the antiviral activity of sanitising agents. Alcohol-based agents cause dissolution of the lipid membrane and denature proteins, thereby disrupting the virus membrane and inhibiting metabolism. The concentration of alcohol in hand-cleansing products, the volume used, contact time, degree of soiling, product formulation and use of excipients are some of the critical factors that affect the efficacy of alcohol against viruses.

Due to its relatively greater lipophilicity, isopropanol is considered more effective than ethanol against SARS-CoV-2. To ensure a greater than 3-log reduction of SARS-CoV-2, a hand sanitiser should ideally contain >80% v/v ethanol or >75% v/v isopropanol. However, recent study which suggests that ethanol and isopropanol used above 30% v/v is effective against SARS-CoV-2 [47] requires confirmation by other investigators. Gel-based hand sanitisers are reported to have more efficacy against enveloped viruses while foam-based preparations have the most rapid drying time. It is recommended that at least 3 ml of product should be used with a total contact time of around 45–50 s. Soiled hands can limit the efficacy of alcohol-based products as well as the presence of excipients; for isopropanol-based formulations, the replacement of glycerol with other emollients is recommended. Similarly, the addition of sodium hydroxide potentiates the antiviral activity of alcohols. Further studies are clearly needed on the optimum design and delivery form of agents for efficient hand decontamination of SARS-CoV-2. Such knowledge will prove of benefit for preparedness against other highly infectious viruses.

## Data Availability

The datasets supporting the conclusions of this review article are included within the article and in references listed in the paper.
